# Comparison of Plug-in Gait and CGM2.3 models reveals systematic differences in joint kinematics and kinetics

**DOI:** 10.1038/s41598-026-52289-x

**Published:** 2026-05-13

**Authors:** Tom Thiel, Anu M. Valtonen, Juha-Pekka Kulmala, Neil Cronin

**Affiliations:** 1 Faculty of Sport and Health Sciences, Neuromuscular Research Center, University of Jyväskylä, Jyväskylä, Finland; 2 Movement Laboratory, Metropolia University of Applied Sciences, Helsinki, Finland; 3 Future Proof Health and Wellbeing Innovation Hub, Metropolia University of Applied Sciences, Helsinki, Finland; 4 Motion Analysis Laboratory, Children’s Hospital, University of Helsinki and Helsinki University Hospital, Helsinki, Finland; 5 JAMK University of Applied Sciences, Jyväskylä, Finland; 6 School of Education and Science, University of Gloucestershire, Gloucester, UK

**Keywords:** Conventional gait model, Kinematics, Kinetics, Gait analysis, Anatomy, Engineering, Health care, Medical research

## Abstract

This study compared two widely used biomechanical models—Plug-in Gait (PiG) and Conventional Gait Model 2.3 (CGM2.3)—during overground walking (WALK) and single-leg squats (SLS) in 24 healthy adults. Data was collected using a 20-camera Vicon system and force plates. Static trials were analyzed with medial knee and ankle markers to align joint axes across models. Kinematic and kinetic outputs were compared using root mean square differences (RMSD) and statistical parametric mapping (SPM) paired t-tests. During WALK, PiG produced greater internal rotation at the knee (RMSD 17.8°, *p* < 0.001) and hip (RMSD 5.0°, *p* < 0.001), and smaller sagittal-plane flexion angles (RMSD 2.6° knee, 2.3° hip) compared with CGM2.3. In single-leg squats, these discrepancies increased to 29.1° and 9.0°, respectively, with sagittal-plane differences of 4.4° at the knee and 5.1° at the hip. CGM2.3 yielded higher knee flexion moments (31% in WALK, 104% in SLS), while PiG produced higher frontal-plane knee moments (28% and 89%). The differences were most pronounced at deeper flexion angles. These results demonstrate that biomechanical outcomes differ systematically between models, emphasizing the impact of model selection on joint kinematics and kinetics in human movement analysis.

**Supplementary Information:**

The online version contains supplementary material available at 10.1038/s41598-026-52289-x.

## Introduction

Biomechanical models provide a framework for analyzing segmental movement during tasks such as walking and squatting. The models also estimate joint loading and muscle function using inverse dynamics^1–3^. However, the choice of coordinate systems can significantly alter kinetic outputs, even within the same model^4–8^. Although reporting guidelines exist^9^, coordinate system details are underreported^10^, hindering comparison across studies.

The Conventional Gait Model (CGM) is one of the most widely used approaches in clinical gait analysis^11^. In Vicon systems, CGM is implemented as Plug-in Gait (PiG), which has contributed substantially to its widespread use in both clinical and research settings. Despite its popularity, PiG has recognized limitations, including inaccurate hip joint centre estimation and poor definition of the femoral coronal plane^12^. To address these limitations, the open-source pyCGM2 framework was developed^3^. While CGM1.0 was designed to reproduce legacy PiG outputs^13^, later versions introduced methodological refinements, including the use of medial knee and ankle markers, a virtual Knee Alignment Device (vKAD) for improved static calibration and tibial torsion estimation, inverse-kinematics-based tracking, and more accurate hip joint centre prediction^14–16^. In addition, CGM2 models default to the Joint Coordinate System (JCS)^17^, in line with International Society of Biomechanics recommendations^9^, which may improve the anatomical interpretability of intersegmental moments^6,8^.

Direct comparisons between PiG and CGM2 remain limited^18–21^. Direct studies have shown better reliability for CGM2 than PiG during running^18^ and treadmill walking^19^ while an abstract-level walking comparison has reported model-dependent differences particularly in frontal- and transverse-plane hip kinematics^21^. A recent cycling study also demonstrated systematic between-model differences and generally better between-day reliability for CGM2^20^. In addition, comparisons across CGM2 variants in typically developing children showed that changes in hip joint centre equations, inverse kinematics, and cluster-based segment tracking altered both gait kinematics and kinetics, with the largest differences generally observed in the transverse plane^22^. Although that study reported only modest between-model differences in walking moments when summarized using RMSD relative to CGM1.0 (a PiG clone), such whole-waveform metrics may obscure localized discrepancies during specific phases of stance. This is important because peak knee moments are commonly used as biomechanically meaningful outcome measures in injury-related movement studies^23,24^.

Nevertheless, further comparisons between PiG and CGM2.3 are warranted, especially in gait, where inconsistent results have been reported. Moreover, it is crucial to investigate tasks involving deep knee flexion, such as single-leg squats (SLS), which involve large joint angles that can induce crosstalk between axes^25,26^, leading to variation in inverse dynamics calculations^27^. Since SLS are commonly used to assess frontal plane knee control and knee abduction moments, which are linked to anterior cruciate ligament (ACL) loading^28,29^, it is essential to understand how these models perform in deep knee flexion tasks.

Therefore, this study aimed to compare the biomechanical outputs of the PiG and CGM2.3 models during walking (WALK) and SLS. Kinematic and kinetic differences were analyzed from the same movement trials, allowing determination of the impact of model selection on joint mechanics. Additionally, the study explored how coordinate system definitions influence kinetic calculations in CGM2.3.

## Methods

All testing was conducted in the Movement Laboratory of Metropolia University of Applied Sciences.

### Participants

Twenty-four healthy and physically active volunteers (12 females, 12 males; age: 28.0 ± 6.0 years). All participants self-reported physical activity levels using the Tegner Activity Scale^30^ averaging 6.3 ± 1.9. Inclusion criteria required participants to be free of lower-limb injuries in the past nine months, and no participants reported a history of lower-limb surgery. The study was approved by the Human Sciences Ethics Committee of the Helsinki Region Universities of Applied Sciences (233/13.02.01/2023) and conducted in accordance with relevant guidelines and regulations, as well as the Declaration of Helsinki. Written informed consent was obtained from all participants.

The sample size was estimated based on a conservative moderate paired effect size (dz = 0.60​) for a two-tailed dependent-means comparison (α = 0.05, power = 0.80). This was the closest available direct PiG vs. CGM2 comparison study at the time of writing that has reported variable-specific paired effects ranging from small to very large in a cycling task^20^.

### Experimental protocol

Participants performed a standardized warm-up including 5 min of stationary cycling at ~ 1.5 W·kg⁻¹, followed by lunges, single-leg deadlifts, and hopping exercises to prepare for the more demanding tasks within the larger data collection protocol. To control for footwear variability, all participants wore standardized indoor court shoes (Asics Gel Rocket 11, Asics Corporation, Kobe, Japan). Movements were performed as follows:

- WALK: Participants walked overground at a target speed of 1.3 m/s, which was monitored using photocells (Witty, Microgate, Italia). The WALK speed was controlled because it has a significant effect on kinematic and kinetic variables^31^. Three valid left-foot contacts were included for analysis, in which only the left foot made complete contact with the force plate without any overlap from the contralateral limb.
- SLS: Participants performed squats on their left leg, with the free leg suspended and hands held together in front. Real-time depth monitoring was conducted using Vicon Nexus software to ensure a consistent squat depth of 90° knee flexion, accompanied by auditory feedback. The participants were instructed to perform the squats as fast as possible while maintaining an adequate range of motion. A single three-repetition trial was used for analysis. The left limb was consistently analyzed to minimize inter-limb variability, which previous studies found to be negligible in healthy populations^32^.

### Data collection

A 20-camera Vicon system (16 Vantage, 4 Vero; Vicon Motion Systems Ltd., Oxford, UK) captured marker trajectories at 300 Hz; eight force plates (AMTI BMS400600, 400 mm × 600 mm) recorded ground reaction forces (GRFs) at 1500 Hz. Three VUE cameras (Vicon) recorded at 100 Hz for qualitative verification, allowing visual confirmation of marker set reconstruction and labeling accuracy, as well as ensuring correct foot placement on the force plates during trials. Data were synchronized and recorded using Vicon Nexus (version 2.14).

Retroreflective markers (9.5 mm diameter) were placed on anatomical landmarks following the CGM2.3 lower-body model, which is backward compatible with the PiG marker set. The CGM2.3 marker set allows the use of vKAD on the PiG model.

### Static calibration

Each participant completed a static calibration trial, which was processed in Vicon Nexus using a standardized pipeline. First, marker trajectories were reconstructed from the infrared camera data and manually labeled to ensure correct marker identification. Anthropometric parameters were then derived from the marker data and used to scale the subject-specific model.

A static skeleton calibration (markers only) was performed and for the PiG model, a Static BodyLanguage Model with a vKAD was used to define knee and ankle joint center locations and axis. This procedure calculates tibial torsion and shank rotation offset using medial knee and ankle markers. The Model also determines anthropometric parameters (LegLength, KneeWidth, AnkleWidth) directly from marker positions. The same parameters were used for both models.

**Fig. 1:**
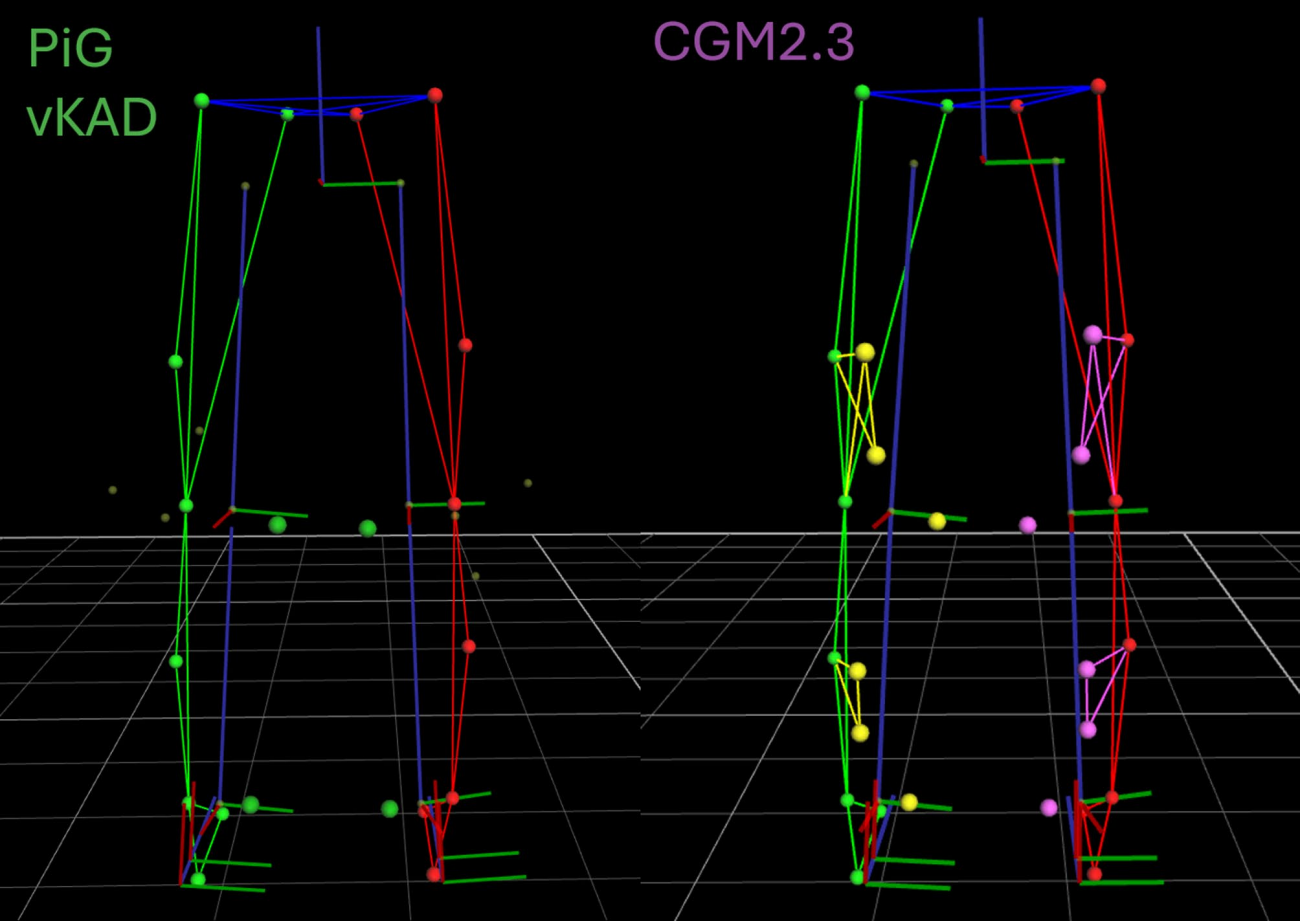
Comparison of marker configurations and models after static calibration. PiG is shown on the left and CGM2.3 on the right.

In the PiG model (v3.3), both feet were assumed to be horizontal during the static trial. For the CGM2.3 model, static calibration (Fig. 1) was performed using the pyCGM2 pipeline with both feet assumed flat.

In the PiG model, the hip joint center is estimated in the pelvic coordinate system using the native regression equations, after which femoral orientation is defined from the hip-to-knee axis and the lateral femoral epicondyle^12^. In CGM2.3 the joint center calculation for hip is done with regression equations by Hara et al.^16^.

The knee joint center for both models was defined as the midpoint of the lateral and medial femoral epicondyle markers. A vKAD calibration was used to define the knee flexion axis and rotational offsets. The tibial orientation was defined from the knee-to-ankle axis and the tibial coronal plane, and the ankle joint center was defined from the malleoli, with the medial malleolar marker used to estimate tibial torsion.

### Dynamic trial processing

For dynamic trials, marker trajectories and GRF data were filtered using a fourth-order, zero-lag Butterworth low-pass filter with a cutoff frequency of 15 Hz^33^.The PiG kinematics and kinetics were calculated using the dynamic PiG model (v 3.3.1) with a distal reaction reference frame (default setting). For the CGM2.3 model, kinematics and kinetics were calculated using the CGM2 framework with JCS projections (default setting).

Additionally, an analysis was performed using the distal coordinate in CGM2.3 to allow for comparison to PiG within the same reference frame. The different coordinate systems have been characterized in previous work^6,8,34^, but in short, the distal and proximal coordinate systems are orthogonal segment-based frames, whereas the JCS expresses moments relative to the joint axes used for the kinematic description. At the knee, the JCS flexion axis follows the proximal segment, the internal–external rotation axis follows the distal segment, and the third axis is a floating axis; therefore, distal and JCS representations are not directly interchangeable in multiplanar tasks^6^.

The kinematic and kinetic model outputs followed a positive rotational direction, where flexion, adduction, and internal rotation were reported as positive^12^. All joint moments were reported as external moments and normalized to body mass.

### Event detection and time normalization

For WALK trials, initial contact and toe-off were defined by vertical GRF crossing a 20 N threshold and the stance phase was time-normalized to 101 points. For SLS, squat descent and ascent events were automatically detected via Left Hip Joint Center (LHJC) vertical-velocity thresholds in Vicon ProCalc (v1.61) and custom Python scripts, with each cycle likewise normalized to 101 time points (0–50% descent, 51–100% ascent).

### Model comparisons

Similarity between the two models was quantified using the root mean square difference (RMSD) over the normalized movement cycle. The RMSD between models was calculated using Eq. (1):

1 $$\:\mathrm{RMSD}=\sqrt{{\frac{1}{n}{\sum\:}_{i=1}^{n}{\left({x}_{i}-{y}_{i}\right)}^{2}}_{}}$$

where x_i_ and y_i_ represent the model outputs at each time point across the normalized movement cycle, and n is the number of time points. Lower RMSD values indicate greater similarity between models, whereas higher values reflect larger overall differences across the movement cycle.

A statistical parametric mapping (SPM) two-tailed paired t-test (*α* = 0.05) was used to compare time series for kinematics and kinetics. SPM analyses were implemented using the open-source spm1d Python library^35^.

To provide a descriptive illustration of the observed between-model differences, hip, knee, and ankle joint center locations were compared between PiG and CGM2.3 for the left limb during SLS in the upright posture and at the deepest squat position. Between-model offsets were calculated as signed global coordinate differences, defined as CGM2.3 minus PiG, in the global X, Y, and Z directions.

These signed offsets are reported as ΔX, ΔY, and ΔZ, respectively, where X represents the anteroposterior direction, Y the mediolateral direction, and Z the superior-inferior direction. The Euclidean distance (d) between the corresponding joint centers was calculated using Eq. (2):

2 $$\:d=\sqrt{{\Delta\:}{X}^{2}+{\Delta\:}{Y}^{2}+{\Delta\:}{Z}^{2}}$$

Positive ΔX, ΔY, and ΔZ values indicate higher coordinate values in CGM2.3 than in PiG along the corresponding global axis.

## Results

**Fig. 2:**
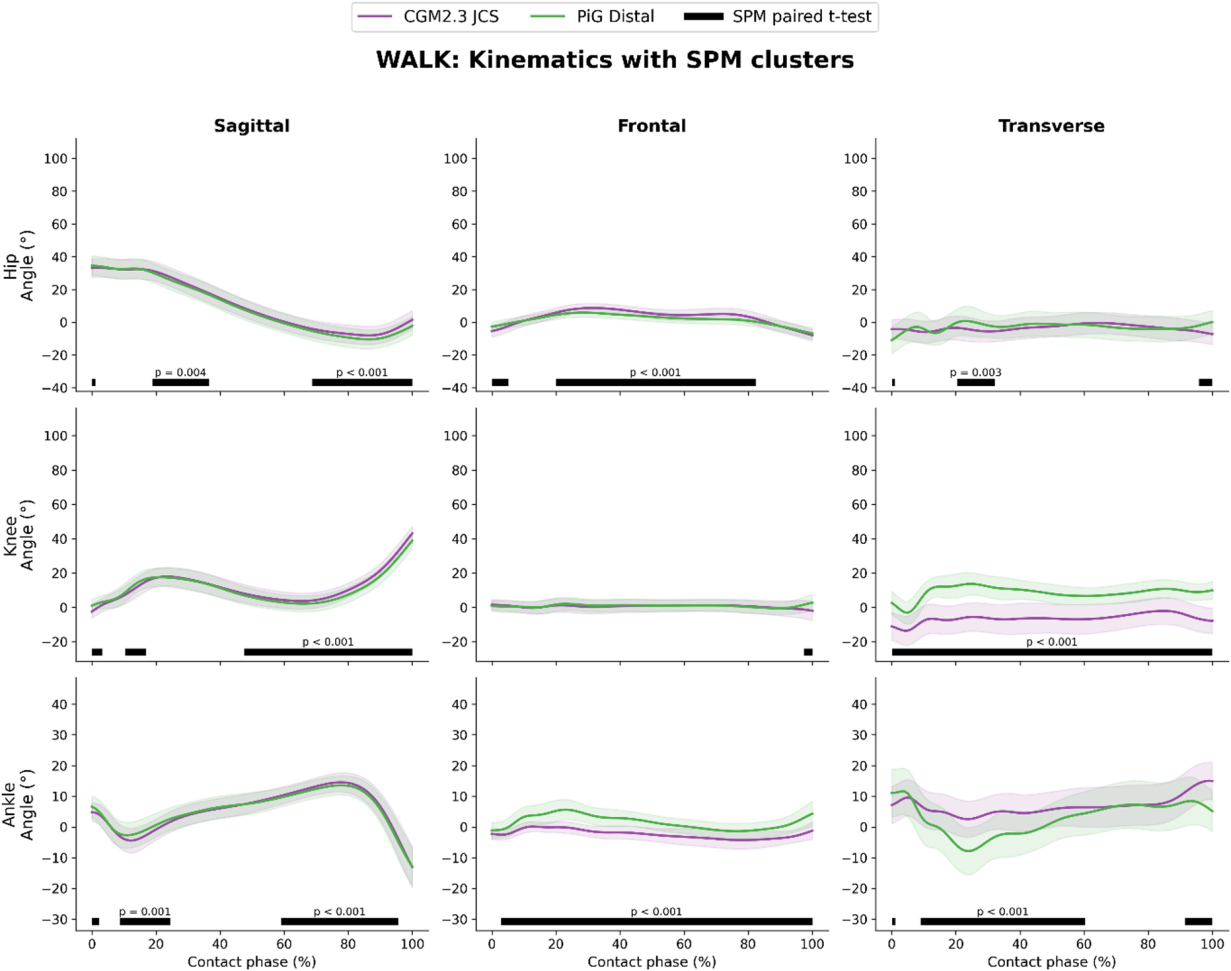
Comparison of WALK kinematics between the CGM2.3 and PiG models using SPM paired t-tests. Statistically significant clusters are highlighted. The lines represent the group mean, and shading denotes the 95% confidence interval. *N* = 72 steps.

**Fig. 3:**
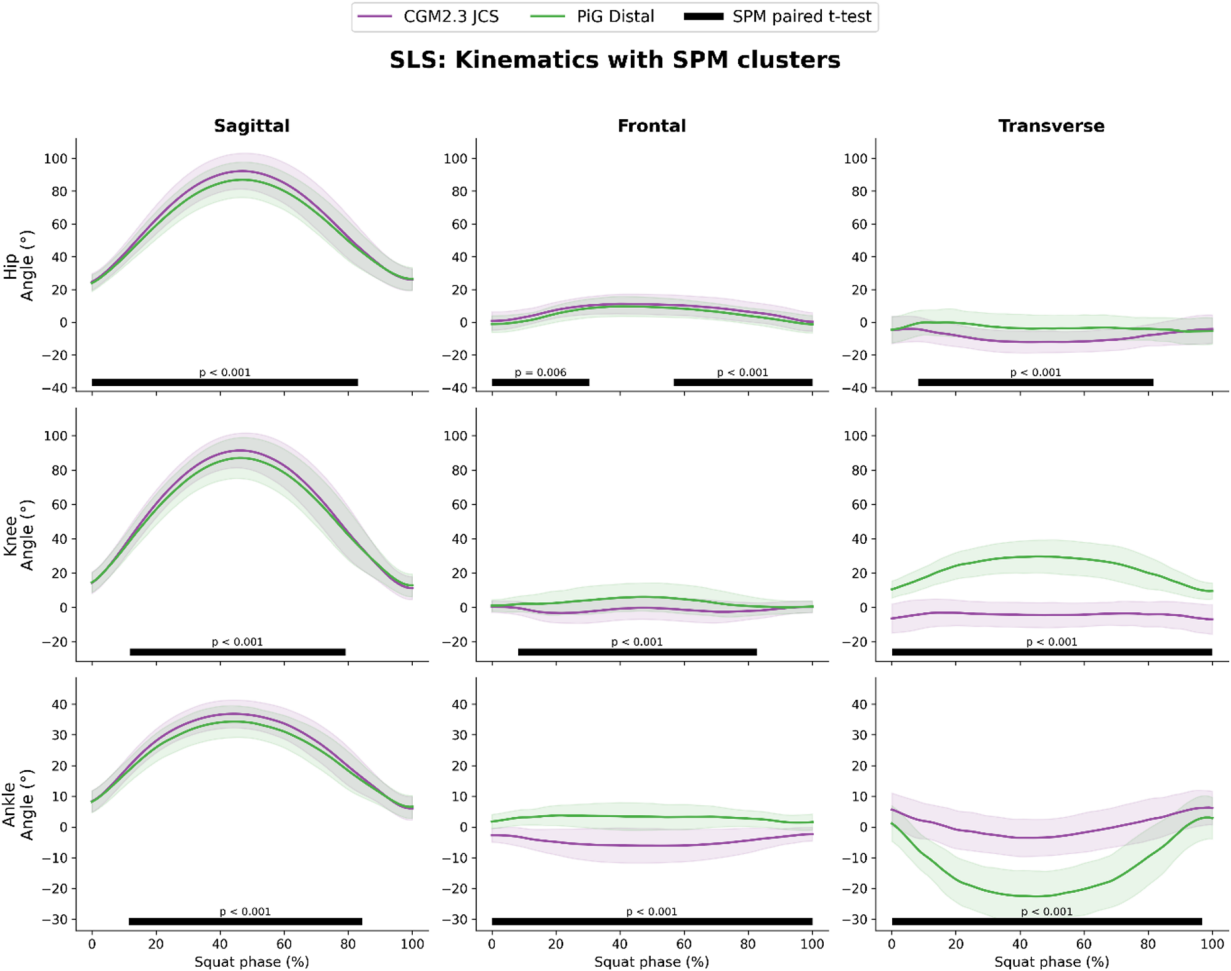
Comparison of SLS kinematics between the CGM2.3 and PiG models using SPM paired t-tests. Statistically significant clusters are highlighted. The lines represent the group mean, and shading denotes the 95% confidence interval. *N* = 72 squats.

**Fig. 4:**
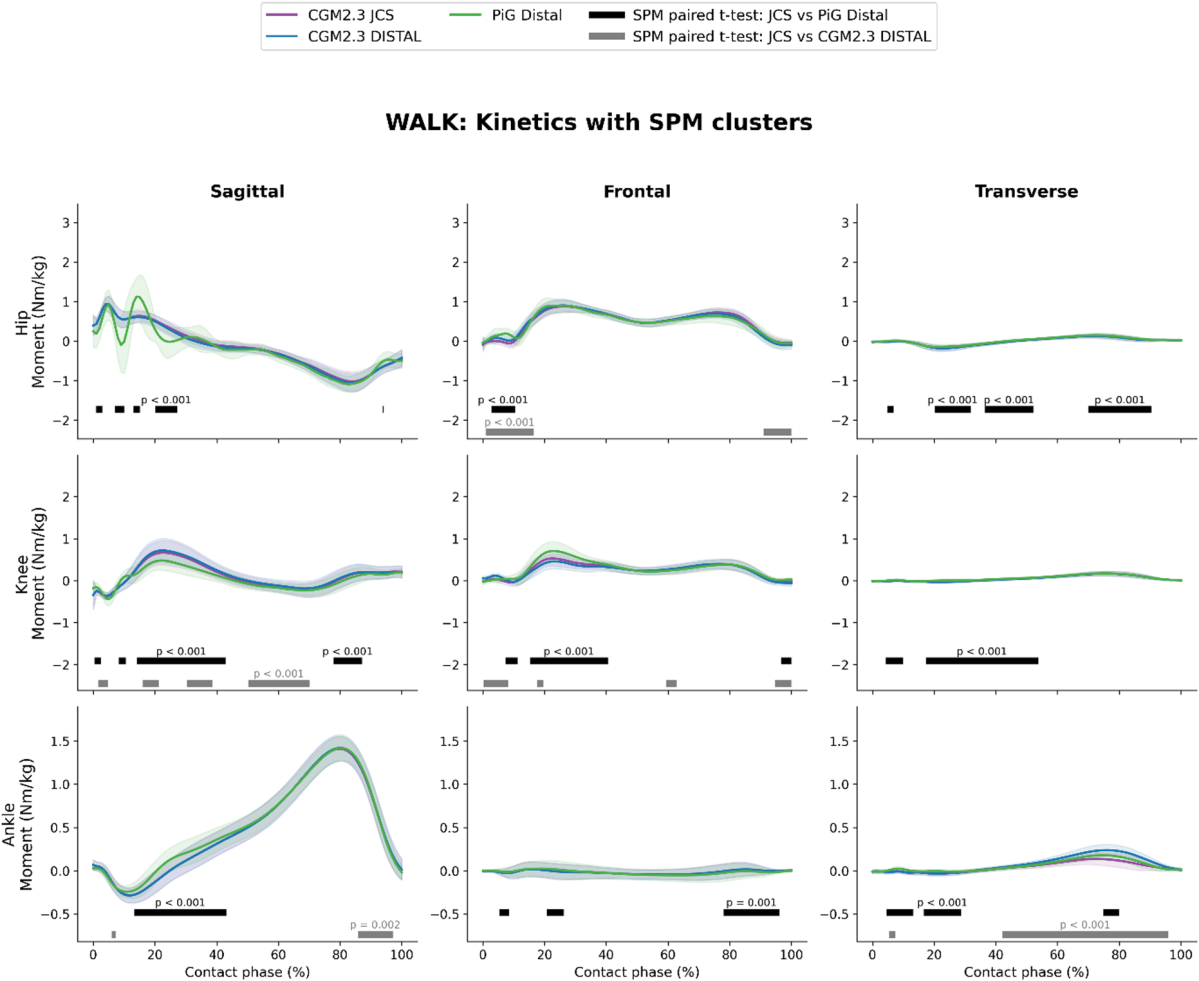
Comparison of WALK kinetics between PiG and CGM2.3 using SPM paired t-tests. Statistically significant clusters are highlighted in black for CGM2.3 JCS and gray for CGM2.3 distal. The lines represent the group mean, and shading denotes the 95% confidence interval. *N* = 72 steps.

**Fig. 5:**
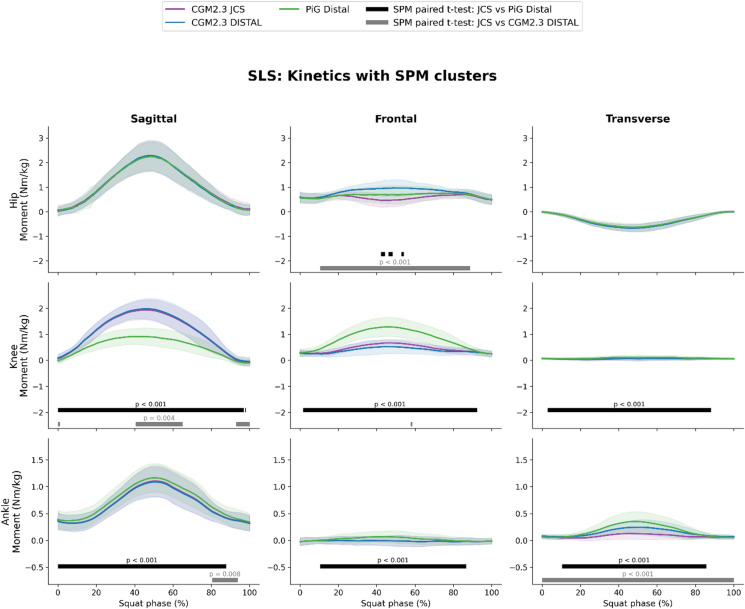
Comparison of SLS kinetics between PiG and CGM2.3 using SPM paired t-tests. Statistically significant clusters are highlighted in black for CGM2.3 JCS and gray for CGM2.3 distal. The lines represent the group mean, and shading denotes the 95% confidence interval. *N* = 72 squats.

SPM results are presented in Figs. 2, 3, 4 and 5. Additional discrete peak values and RMSD comparisons, coordinate-system comparisons, segment-length calculations, and joint-center analyses are provided in the Supplementary Information (Appendices A–D).

### WALK kinematics

During WALK, hip kinematics differed between models in all three planes. In the sagittal plane, hip peak flexion values were similar between models (34.69° vs. 33.59°, RMSD 2.27°), but SPM clusters indicated significant statistical differences particularly during mid-stance (*p* = 0.004) and pre-swing (*p* < 0.001). In the frontal plane, CGM2.3 yielded greater hip adduction angles than PiG (8.97° vs. 6.00°), with an RMSD of 3.27° and a significant SPM cluster was found during mid-stance. The transverse plane also showed a clear model-related discrepancy, with PiG producing more internally rotated hip angles, and an RMSD of 5.03°.

At the knee, CGM2.3 yielded consistently higher peak flexion angles than PiG during walking (43.08° vs. 38.87°), with an RMSD of 2.61°. Significant SPM clusters were found at early stance (*p* < 0.001) and late stance phases (*p* < 0.001). In the frontal plane, PiG produced greater adduction angles and the RMSD was 2.31°. In the transverse plane, large systematic differences were observed, with PiG producing more internal rotation throughout the movement. The RMSD was 17.78°, and the SPM analysis showed a significant (*p* < 0.001) cluster spanning the entire contact phase.

At the ankle, significant WALK kinematic differences were also observed in all three planes. In the sagittal plane the RMSD value was just 1.31°, but the SPM clusters were present during early contact (*p* = 0.001) and again during the late contact phase (*p* < 0.001). In the frontal plane, a significant cluster extended across nearly the entire contact phase (*p* < 0.001), indicating a consistent offset between the models with RMSD of 4.20°. In the transverse plane, significant differences were observed during early-to-mid contact (*p* < 0.001), with an additional brief cluster at the end of the contact phase, with RMSD value of 4.28°.

### SLS kinematics

During SLS, the sagittal-plane hip flexion angles differed significantly between models (93.09° vs. 87.85°, RMSD 5.09°). SPM clusters indicated significant statistical differences throughout the movement (*p* < 0.001). In the frontal plane, CGM2.3 yielded greater hip adduction angles than PiG (12.61° vs. 10.84°), with an RMSD of 3.43°. Significant SPM clusters were found during both the descending (*p* = 0.006) and ascending (*p* < 0.001) phases. The transverse plane showed the largest hip-related model discrepancies, with PiG producing more internally rotated hip angles throughout the movement. The RMSD was 8.96°, and SPM clusters indicated systematic differences throughout the movement (*p* < 0.001).

At the knee, CGM2.3 yielded consistently higher peak flexion angles than PiG during SLS (92.36° vs. 88.06°), with an RMSD of 4.37°. Significant SPM clusters were present across nearly the entire SLS cycle (*p* < 0.001). In the frontal plane, PiG produced greater adduction angles, whereas CGM2.3 showed more abducted positions. Peak knee adduction was higher in PiG (7.59° vs. 2.62°), and the RMSD was 6.95°. In the transverse plane, large systematic differences were again observed, with PiG producing more internal rotation throughout the movement. The RMSD was 29.15°, and the SPM analysis showed significant (*p* < 0.001) clusters extending over most of the task duration.

Ankle kinematics during SLS also differed systematically between models. In the sagittal plane, significant differences were observed throughout the squat cycle (*p* < 0.001), with CGM2.3 showing consistently greater dorsiflexion than PiG. In the frontal plane, a significant cluster extended across the whole movement (*p* < 0.001), indicating a persistent between-model offset. The transverse plane likewise showed large systematic differences throughout the squat cycle (*p* < 0.001) with RMSD value 15.27°.

A descriptive supplementary analysis of modeled segment lengths showed that hip–knee and knee–ankle distances remained unchanged between upright and deepest squat positions in CGM2.3, whereas both distances decreased in PiG, particularly at the deepest squat position (Supplementary Table S3 and Fig. S5).

### WALK kinetics

During WALK, hip kinetics showed clear deviations in all planes, with the largest differences in the sagittal plane, where PiG produced 49% higher peak moments and exhibited distinct spikes during early stance. In the frontal and transverse planes, SPM paired t-tests (black bars) also revealed significant differences (*p* < 0.001) during discrete parts of the contact phase.

Regarding knee kinetics in the sagittal plane, CGM2.3 yielded higher joint moments, with 31% greater peak values during WALK compared to PiG. In contrast, PiG produced higher frontal plane peak knee moments, with a difference of 28% in WALK. The transverse plane differences were minor, with PiG showing 6% higher peak internal rotation moment during WALK.

At the ankle, the clearest kinetic difference during walking was observed in the sagittal plane, where significant differences were present during early-to-mid contact (*p* < 0.001). In the frontal plane, ankle moments were small in magnitude, but significant clusters were still present in early contact and again in late stance (*p* = 0.001). In the transverse plane, moments likewise remained small, although significant clusters were observed during early contact and again during mid-to-late stance (both *p* < 0.001). Additional gray-bar clusters indicated that reprojecting CGM2.3 into distal axes did not eliminate these ankle moment differences. Discrete peak values and RMSD comparisons are provided in Supplementary Tables S1 and S2.

To assess the impact of coordinate system definitions, a comparison was made between PiG and CGM2.3 distal, with both models using distal projections. Unexpectedly, greater kinetic discrepancies were observed. Gray bars in Figs. 4 and 5 highlight regions with statistically significant differences, often matching or exceeding those seen with JCS. The comparison of CGM2.3’s JCS and distal projections showed identical hip and knee rotation profiles, and small sagittal plane differences. However, small but significant frontal plane clusters emerged during WALK and SLS (Supplementary B, Figs. S1 and S3).

### SLS kinetics

During SLS, hip kinetics showed good agreement between models in the sagittal and transverse planes. However, in the frontal plane, CGM2.3 JCS yielded higher abduction moments (*p* < 0.001) than both PiG and CGM2.3 distal (gray bars), especially during phases of greater hip flexion.

Regarding knee kinetics, CGM2.3 yielded higher joint moments in the sagittal plane, with 104% greater peak values during SLS compared to PiG. In contrast, PiG produced higher frontal plane peak knee moments, with a difference of 89% in SLS. The transverse plane differences were minor, with PiG showing 33% higher peak internal rotation moment during SLS. SPM paired t-tests (black bars) revealed significant differences (*p* < 0.001) across all planes throughout the entire movement cycle, highlighting systematic variation between models.

At the ankle, significant between-model differences were observed in all three planes during SLS. In the sagittal plane, ankle moments differed throughout most of the movement (*p* < 0.001), with PiG generally showing higher values than CGM2.3. In the frontal plane, the absolute moment magnitudes remained close to zero, but significant differences were still detected across the movement (*p* < 0.001). In the transverse plane, significant differences were observed throughout the squat cycle (*p* < 0.001), with PiG showing the largest moments, CGM2.3 distal intermediate values, and CGM2.3 JCS the smallest. Gray bars further indicated that coordinate system projection explained only part of these ankle kinetic discrepancies.

## Discussion

This study showed that PiG and CGM2.3 are not interchangeable for lower-limb kinematics and kinetics during WALK and SLS. The main kinematic finding was that PiG produced systematically more internal rotation and lower sagittal-plane flexion at the hip and knee than CGM2.3, with the largest between-model differences observed in transverse-plane knee kinematics (RMSD 17.78° during WALK and 29.15° during SLS) and in sagittal-plane flexion during deep squat positions (hip: 87.85° vs. 93.09°; knee: 88.06° vs. 92.36°). Frontal-plane differences were smaller but consistent, with CGM2.3 showing greater hip adduction and PiG showing greater knee adduction. Ankle kinematics also differed significantly between models in all planes, particularly during SLS, where the transverse-plane RMSD reached 15.27°. The main kinetic finding was that CGM2.3 yielded larger knee flexion moments, whereas PiG yielded larger frontal-plane knee moments. Based on peak values, knee flexion moments were 31% higher in CGM2.3 during WALK (0.68 vs. 0.52 Nm/kg) and 104% higher during SLS (1.98 vs. 0.97 Nm/kg), whereas PiG produced 28% higher frontal-plane knee moments during WALK (0.73 vs. 0.57 Nm/kg) and 89% higher values during SLS (1.32 vs. 0.70 Nm/kg). At the hip, the largest kinetic discrepancy was observed during early stance in WALK, where PiG produced 49% higher sagittal-plane peak moments (1.46 vs. 0.98 Nm/kg), while hip moments during SLS were much more similar between models except in the frontal plane during deep flexion. Importantly, reprojecting CGM2.3 moments into distal axes did not eliminate the between-model differences, indicating that coordinate-system choice explained only part of the observed kinetic discrepancy.

### Kinematic differences

The clearest kinematic difference during WALK was the greater internal rotation at the knee produced by PiG. During WALK, PiG showed more internally rotated hip and knee angles than CGM2.3, with the largest difference at the knee (RMSD 17.78°, *p* < 0.001 over most of stance), while the hip transverse-plane RMSD was 5.03° and likewise remained significantly different (*p* < 0.001). These findings are consistent with previous reports that transverse-plane outputs are among the most model-sensitive variables in the CGM family^22^, and with previous observations of model-dependent hip kinematics between CGM2.3 and PiG in WALK^21^. They are also biomechanically plausible, because PiG is known to be sensitive to lower-limb marker placement, especially in the non-sagittal planes^36^, and because conventional direct-kinematic implementations are generally more vulnerable to rotational error than cluster-based inverse-kinematics approaches^37,38^. In practical terms, the present results indicate that even when sagittal-plane gait curves appear broadly similar between models, transverse-plane hip and especially knee angles may differ enough to affect interpretation.

The most important new kinematic observation was that between-model differences increased markedly as flexion increased during SLS, and the additional joint center analysis (Supplementary Table S4) provides quantitative support for this interpretation. At the deepest squat position, the Euclidean distance between the models’ joint centers increased from 22.33 to 30.67 mm at the hip, from 9.92 to 41.81 mm at the knee, and from 8.67 to 20.68 mm at the ankle. At the same time, modeled segment lengths remained unchanged in CGM2.3 but shortened in PiG, with the thigh-length difference increasing from 21.44 to 55.10 mm and the shank-length difference from − 0.04 to 30.32 mm between upright and deepest squat positions. Most notably, the knee joint center in CGM2.3 was located 40.02 mm anterior to the PiG knee joint center at the deepest SLS position (Supplementary Fig. S6). Together, these findings support the idea that the greater flexion angles seen in CGM2.3 are linked to systematic between-model changes in joint center location and effective segment geometry during deep flexion.

The sagittal- and frontal-plane kinematic differences also appear to reflect known structural limitations of PiG. During SLS, PiG yielded lower hip and knee flexion, greater knee adduction, and more internal rotation than CGM2.3, with significant differences extending through almost the entire movement cycle (all major SPM clusters *p* < 0.001). This pattern is compatible with previous work showing that conventional marker-based lower-limb models can suffer from femoral frontal-plane definition errors and kinematic cross-talk, particularly when knee flexion is large^12,25,26^. It is also relevant that tibial torsion handling differs between older PiG implementations and later CGM2 developments^3^, and that PiG follows a more hierarchical top-down framework, meaning that proximal definition errors can propagate distally^38^. The difference in hip joint centre estimation may also contribute, because CGM2.3 uses the Hara et al.^16^ formulation whereas PiG relies on the older Davis et al.^1^ method. For these reasons, the observed kinematic differences should not be attributed to one single factor. Rather, the present data suggest that joint center location, segment-length behaviour, frontal-plane femur definition, tibial torsion treatment, and susceptibility to soft-tissue artifacts (STA) all act together to produce the between-model differences, especially in deep flexion.

Ankle kinematics also differed systematically between models, which indicates that the consequences of model choice are not limited to the hip and knee. During WALK, ankle differences were present in all three planes, with RMSD values of 1.31° in the sagittal plane, 4.20° in the frontal plane, and 4.28° in the transverse plane, while during SLS the largest discrepancy was observed in transverse-plane ankle kinematics (RMSD 15.27°, *p* < 0.001 throughout the cycle). Although the ankle is a distal joint, these findings are consistent with the idea that proximal modelling choices can alter downstream segment orientation and joint center definition. The ankle-center difference at the deepest squat position was 20.68 mm, including a -15.36 mm superior-inferior offset, which further supports the interpretation that deep-flexion geometry differs substantially between models.

### Kinetic differences

The clearest kinetic difference during walking was the atypical sagittal-plane hip moment pattern produced by PiG. During WALK, PiG generated 49% higher peak sagittal-plane hip moments than CGM2.3 (1.46 vs. 0.98 Nm/kg) and showed pronounced spikes during early stance, while significant differences were also present in the frontal and transverse planes (*p* < 0.001). These early-stance fluctuations were not observed in CGM2.3 despite identical filtering procedures, suggesting that the discrepancy is primarily related to model characteristics rather than to a simple processing artifact. Proximal joint moments are especially sensitive to joint segment accelerations^1^ and joint center estimation has been emphasized as one of the most influential factors affecting inverse-dynamics moments^10^. Accordingly, the observed hip kinetic differences are most likely related to differences in joint center estimation and segment orientations of the two approaches. In addition, PiG may be more susceptible to STA, which most likely contributes to segment length variability and the irregular hip moment pattern. Although filtering choices are known to influence inverse-dynamics peaks, particularly in dynamic tasks^33^. Only one cutoff frequency (15 Hz) was used in the present study. Thus, the current data does not allow direct conclusions regarding filtering sensitivity, but the absence of similar fluctuations in CGM2.3 suggests that PiG may be more vulnerable to such effects.

The most clinically relevant kinetic difference was the consistent underestimation of knee sagittal-plane moments by PiG relative to CGM2.3. During WALK, CGM2.3 produced 31% higher knee flexion moments than PiG, and during SLS this difference increased to 104%. At the same time, PiG produced higher frontal-plane knee moments by 28% during WALK and 89% during SLS, while transverse-plane kinetic differences remained smaller in magnitude. This result is important because it shows that the between-model discrepancy is not simply a scaling issue: the relative loading profile shifts between planes. The finding is also consistent with the kinematic results, because the largest kinetic differences were observed precisely in phases where the models differed most in flexion angle, joint center locations and segment lengths. The present data therefore support the interpretation that PiG’s lower knee flexion moments are linked to its lower knee flexion angles and altered segment lengths in deep flexion. This interpretation is strengthened by the supplementary segment-length and joint center results and is also compatible with the recent finding by Kulmala^39^, who reported that sagittal-plane knee moments from Vicon’s PiG were 27% lower than those measured using an isokinetic dynamometer. Together, these findings suggest that PiG may systematically underestimate sagittal-plane knee loading during tasks involving substantial flexion.

The present results also clarify how whole-waveform similarity and peak-value agreement can lead to different conclusions. Kerr et al.^22^ reported relatively modest gait-moment RMSD values between CGM1.0 and CGM2 variants, which might suggest acceptable agreement at first glance. However, the present study showed that localized peak differences can still be substantial even when waveform-level differences appear moderate. This is particularly relevant for injury-related and performance-related studies, where discrete peak knee moments are often used as primary outcome variables. Accordingly, RMSD alone should not be interpreted as evidence of interchangeability on its own when the scientific question depends on specific phases of stance or peak values.

Coordinate system definitions are known to affect joint kinetic outputs, but their contribution to between-model differences is not always addressed explicitly^6,7,10,34^. In the present comparison, CGM2.3 used JCS, whereas PiG used distal projection. Although the representations are known to differ in their outputs^10,27,34^, their specific role in explaining kinetic disagreement between models remains unclear.

To examine this, an additional analysis was performed in which joint moments from both models were expressed using distal projection. Contrary to expectation, this did not reduce the between-model differences; in some regions, the differences became larger. This suggests that the observed discrepancies cannot be explained by coordinate system choice alone, but are more likely driven by broader differences in model definition and implementation.

Within CGM2.3, the comparison between JCS and distal projection showed near-identical moment profiles in the sagittal and transverse planes. Small but statistically significant differences remained in the frontal plane, particularly during WALK at initial contact and toe-off, and during SLS under deeper flexion (Supplementary Figs. S1 and S3). This pattern is consistent with previous work showing that coordinate system selection can influence the interpretation of hip and knee moments, especially outside the sagittal plane^7,10^.

During SLS, agreement between the two CGM2.3 representations remained high overall, with the main within-model difference again observed in the frontal-plane hip moment, becoming more pronounced as flexion increased (Supplementary Fig. S3). Taken together, these findings indicate that coordinate system selection had a measurable but limited effect in the present dataset, whereas the larger PiG–CGM2.3 differences primarily reflect other model-related factors.

The ISB recommends the use of JCS for intersegmental moments^9^ because it provides an anatomically interpretable representation of joint loading and muscle function^6^. The present findings support that recommendation, indicating that careful reporting of both model type and coordinate representation is essential.

## Limitations

This study has several limitations. The sample consisted of 24 healthy adults, and only the left limb was analyzed, which limits generalizability to older individuals or clinical populations. In addition, the analysis was based on only three valid walking contacts and a single three-repetition SLS trial, which may have increased the influence of within-subject variability, particularly for discrete peak values. Only one WALK speed (1.3 m/s) and one standardized SLS condition were tested, so the findings should be considered task- and condition-specific. The additional joint center and segment-length analysis was descriptive and limited to the upright and deepest SLS positions rather than the full time series, and therefore cannot determine the exact temporal mechanism by which between-model geometric differences affected the waveforms. In addition, joint center orientations were not analyzed separately, although they are likely relevant for both the kinematic and kinetic differences observed between models. Deep flexion was associated with a marked anterior shift in the modeled knee joint center, particularly in CGM2.3 relative to PiG, which may have contributed to the reduced flexion angles observed in PiG. However, the present study cannot determine whether the lower knee moments in PiG were caused directly by reduced knee flexion, altered moment-arm geometry, differences in effective segment length, or potential differences in the location of the segment centers of mass. Furthermore, both WALK and SLS data were filtered using a single low-pass cutoff frequency of 15 Hz. Although this standardized processing enabled direct comparison between the models, it remains unclear whether the observed hip moment fluctuations in PiG were influenced by filtering sensitivity, which was not examined in the present study. Finally, the comparison was made between specific software implementations of PiG and CGM2.3 rather than against a direct in vivo reference. Both models remain marker-based representations of human movement and are affected by STA, joint center estimation error, and assumptions regarding segment geometry and joint mechanics. Thus, the present findings demonstrate how model choice influences kinematic and kinetic outputs derived from the same dataset, but they do not establish which model is more anatomically accurate in an absolute sense.

## Conclusions

In conclusion, PiG and CGM2.3 produced systematically different lower-limb kinematics and kinetics during both WALK and SLS, with the largest discrepancies occurring in transverse-plane rotations and in tasks involving deeper flexion. The joint center and segment-length analyses indicate that these differences are at least partly explained by modeled joint geometry during squat depth, especially at the knee. Kinetic differences, particularly the lower knee flexion moments and higher frontal-plane knee moments in PiG, were not resolved by matching coordinate-system projections, indicating that they arise mainly from broader model-definition differences. These findings highlight that model choice is not a minor technical detail but a substantive methodological decision that can affect interpretation of joint mechanics, comparison across studies, and the clinical meaning. Future research should further investigate the clinical implications of these modeling differences, particularly in high-impact and dynamically loaded conditions.

## Electronic Supplementary Material

Below is the link to the electronic supplementary material.

Supplementary Material 1.

## Data Availability

The datasets generated during and/or analysed during the current study are available from the corresponding author on reasonable request.
