# Supplementary material for: Comparison of Plug-in Gait and CGM2.3 models reveals systematic differences in joint kinematics and kinetics
Source: Sci Rep. 2026 May 13;16:21805. doi: 10.1038/s41598-026-52289-x (PMC13357812; doi:10.1038/s41598-026-52289-x)
Supplement: Supplementary file 1 — Supplementary Material 1. [file 41598_2026_52289_MOESM1_ESM.docx]

**Supplementary Information**

**Comparison of Plug-in Gait and CGM2.3 models reveals systematic differences in joint kinematics and kinetics**

Tom Thiel, Anu M Valtonen, Juha-Pekka Kulmala, Neil Cronin

Table of Contents

**Supplementary Appendix A. Peak values and RMSD comparisons2**

Supplementary Table S1. Kinematic peak values and RMSD comparisons 2

Supplementary Table S2. Kinetic peak values and RMSD comparisons3

**Supplementary Appendix B. Coordinate-system comparisons** 4

Supplementary Fig. S1. CGM2.3 JCS and distal coordinate systems on WALK4

Supplementary Fig. S2. CGM2.3 JCS and proximal coordinate systems on WALK5

Supplementary Fig. S3. CGM2.3 JCS and distal coordinate systems on SLS6

Supplementary Fig. S4. CGM2.3 JCS and proximal coordinate systems on SLS7

**Supplementary Appendix C. Segment-length analysis**8

Supplementary Table S3. Segment-length differences8

Supplementary Fig. S5. Segment-length differences8

**Supplementary Appendix D. Joint-center analysis**9

Supplementary Table S4. Joint-center location differences9

Supplementary Fig. S6. Joint-center positions10

# Appendix A

|  |  | **WALK** | | | **SLS** | | |
| --- | --- | --- | --- | --- | --- | --- | --- |
|  |  | **Peak values (range)** | | **RMSD** | **Peak values (range)** | | **RMSD** |
|  |  | **CGM2.3** | **PiG** |  | **CGM2.3** | **PiG** |  |
| **Joint** | **Plane** | **degrees** | **degrees** | **degrees** | **degrees** | **degrees** | **degrees** |
| **Hip** | Sagittal | 33.59 (21.94 - 43.35) | 34.69 (21.13 - 44.90) | 2.17 | 93.09 (73.58 - 111.45) | 87.85 (68.61 - 102.73) | 4.28 |
|  | Frontal | 8.97 (3.06 - 13.05) | 6.00 (-0.26 - 10.37) | 3.08 | 12.61 (1.41 - 23.52) | 10.84 (0.02 - 21.28) | 2.85 |
|  | Transversal | 1.10 (-12.83 - 14.10) | 5.16 (-7.36 - 19.58) | 4.83 | -2.17 (-18.83 - 10.27) | 2.90 (-13.08 - 20.07) | 7.98 |
| **Knee** | Sagittal | 43.08 (34.88 - 50.57) | 38.87 (28.82 - 47.81) | 2.53 | 92.36 (75.06 - 115.37) | 88.06 (60.38 - 111.98) | 3.73 |
|  | Frontal | 3.05 (-2.92 - 10.20) | 4.53 (-1.12 - 11.35) | 2.17 | 2.62 (-6.36 - 11.31) | 7.59 (-4.18 - 19.66) | 6.04 |
|  | Transversal | -1.22 (-13.10 - 11.03) | 15.77 (4.59 - 27.47) | 15.62 | -0.66 (-15.03 - 11.20) | 31.02 (11.94 - 53.77) | 27.72 |
| **Ankle** | Sagittal | 14.90 (5.63 - 18.96) | 13.96 (4.67 - 18.52) | 1.31 | 37.21 (25.82 - 48.21) | 34.80 (23.22 - 44.13) | 2.9 |
|  | Frontal | 1.01 (-3.68 - 6.04) | 6.28 (-2.00 - 12.52) | 4.2 | -1.58 (-6.08 - 5.34) | 5.57 (1.15 - 13.35) | 8.04 |
|  | Transversal | 15.40 (3.48 - 24.00) | 13.70 (-3.50 - 23.99) | 6.21 | 7.66 (-5.47 - 16.12) | 4.32 (-8.57 - 16.08) | 15.27 |

Supplementary Table S1. Kinematic peak values of CGM2.3 and PiG and RMSD values of the whole stance phase, between the models in WALK and SLS. The peak values are presented as the average peak value, and the lowest and highest values are in parentheses. N = 72 steps N = 72 squats.

| **as** |  |  | | |  | | |
| --- | --- | --- | --- | --- | --- | --- | --- |
|  |  | **WALK** | | | **SLS** | | |
|  |  | **Peak values (range)** | | **RMSD** | **Peak values (range)** | | **RMSD** |
|  |  | **CGM2.3** | **PiG** |  | **CGM2.3** | **PiG** |  |
| **Joint** | **Plane** | **Nm/kg** | **Nm/kg** | **Nm/kg** | **Nm/kg** | **Nm/kg** | **Nm/kg** |
| **Hip** | Sagittal | 0.98 (0.63 - 1.31) | 1.46 (0.70 - 2.21) | 0.31 | 2.35 (1.33 - 3.45) | 2.34 (1.29 - 3.40) | 0.16 |
|  | Frontal | 0.91 (0.69 - 1.20) | 0.96 (0.67 - 1.31) | 0.14 | 0.84 (0.59 - 1.07) | 0.93 (0.51 - 1.23) | 0.2 |
|  | Transversal | 0.13 (0.02 - 0.21) | 0.15 (0.05 - 0.21) | 0.02 | 0.01 (-0.09 - 0.09) | 0.01 (-0.08 - 0.09) | 0.05 |
| **Knee** | Sagittal | 0.68 (0.23 - 1.16) | 0.52 (0.16 - 0.81) | 0.13 | 1.98 (1.31 - 2.89) | 0.97 (0.40 - 1.57) | 0.66 |
|  | Frontal | 0.57 (0.29 - 0.88) | 0.73 (0.44 - 1.28) | 0.09 | 0.70 (0.35 - 0.94) | 1.32 (0.74 - 2.04) | 0.43 |
|  | Transversal | 0.17 (0.08 - 0.24) | 0.18 (0.09 - 0.23) | 0.02 | 0.12 (0.03 - 0.23) | 0.16 (0.06 - 0.32) | 0.04 |
| **Ankle** | Sagittal | 1.42 (1.11 - 1.71) | 1.42 (1.08 - 1.66) | 0.07 | 1.14 (0.74 - 1.82) | 1.20 (0.85 - 1.88) | 0.08 |
|  | Frontal | 0.06 (0.00 - 0.24) | 0.06 (-0.00 - 0.28) | 0.02 | 0.06 (-0.05 - 0.20) | 0.11 (-0.05 - 0.30) | 0.05 |
|  | Transversal | 0.14 (0.03 - 0.29) | 0.18 (0.07 - 0.28) | 0.04 | 0.17 (0.06 - 0.43) | 0.38 (0.13 - 0.74) | 0.13 |

Supplementary Table S2. Kinetic peak values of CGM2.3 JCS and PiG and RMSD values of the whole movement between the models in WALK and SLS. The peak values are presented as the average peak value. and the lowest and highest values are in parentheses. N = 72 steps N = 72 squats.

# Appendix B


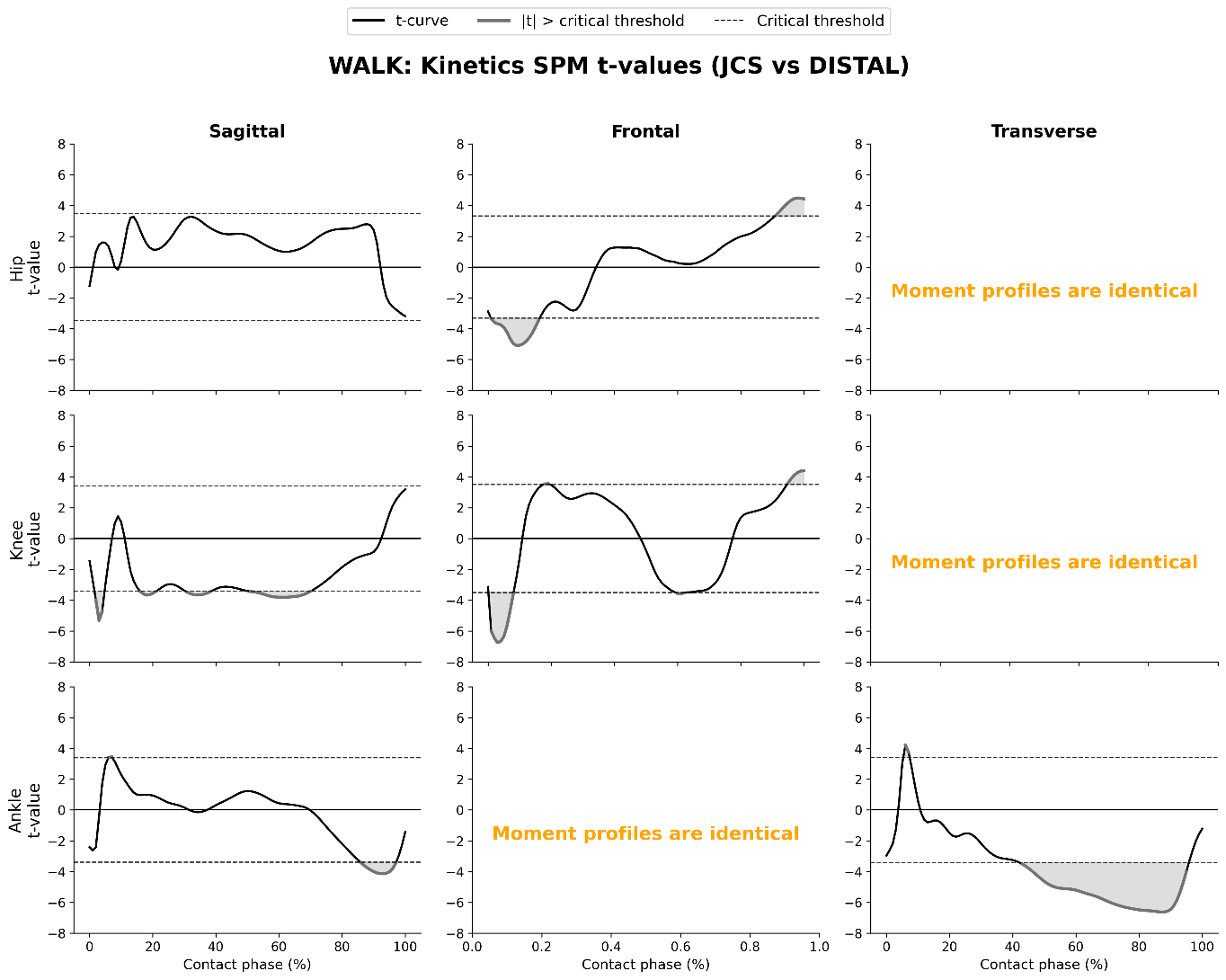


Supplementary Fig. S1. Comparison of CGM2.3 JCS and distal coordinate systems on WALK.


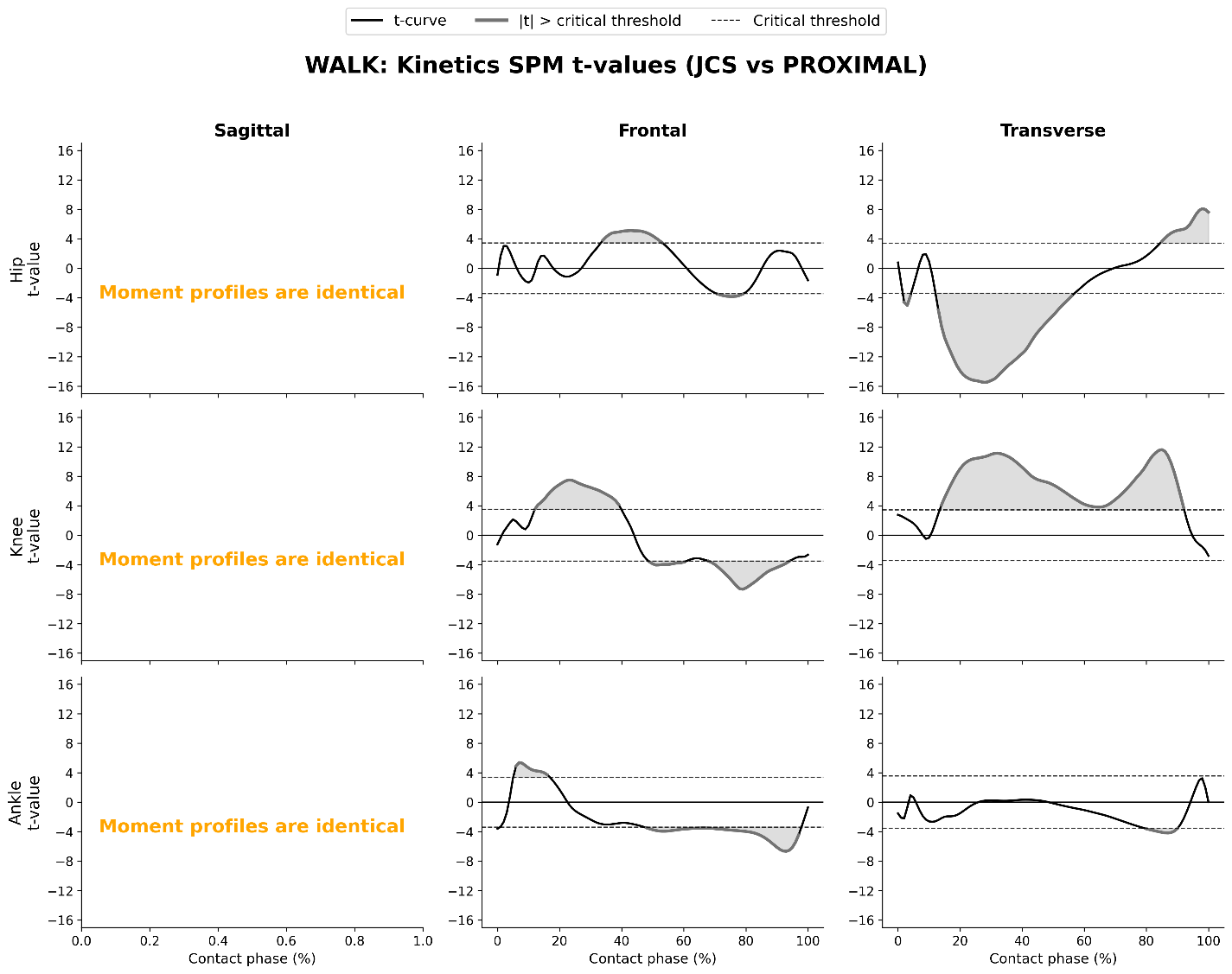
Supplementary Fig. S2. Comparison of CGM2.3 JCS and proximal coordinate systems on WALK.


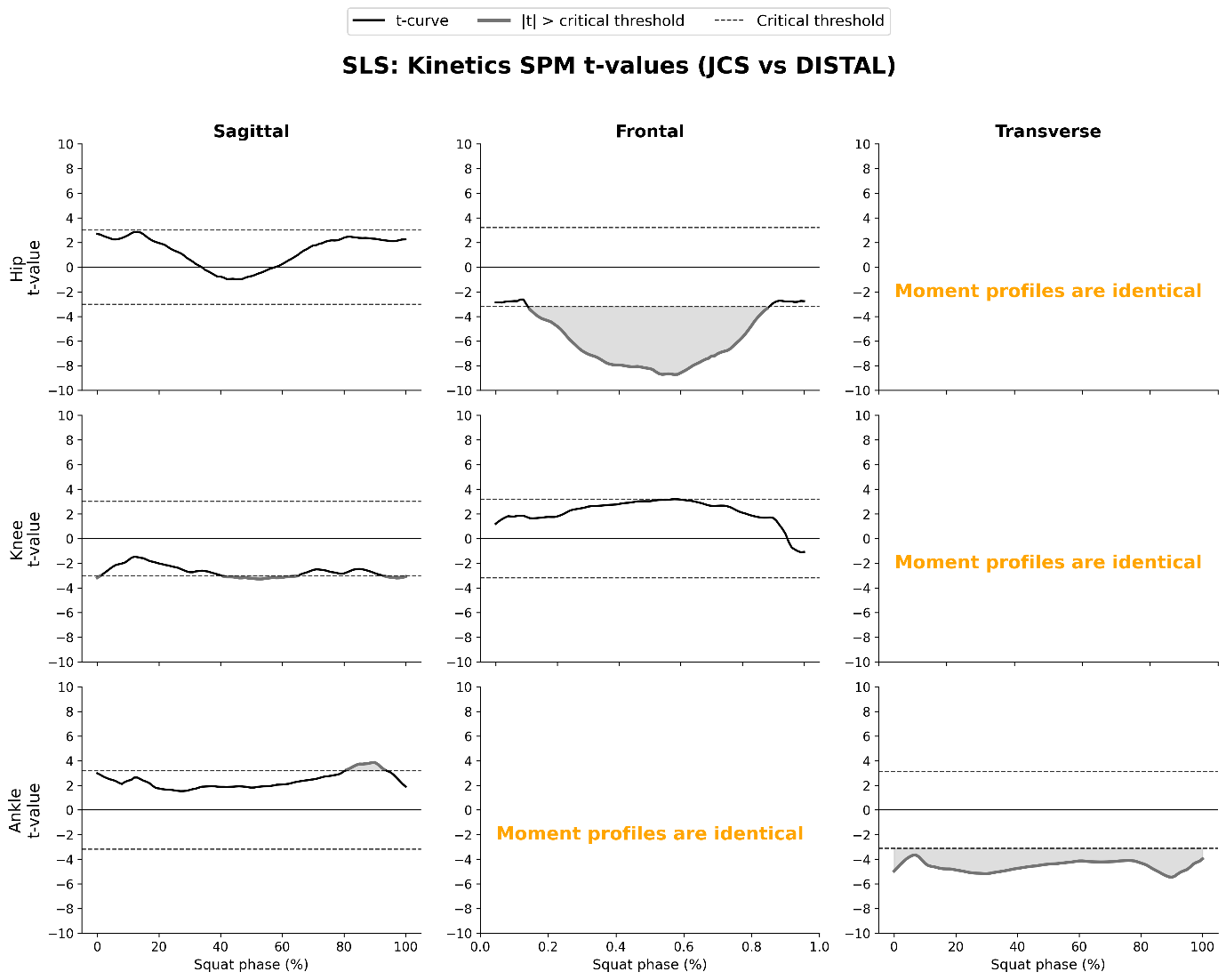


Supplementary Fig. S3. Comparison of CGM2.3 JCS and distal coordinate systems on SLS.


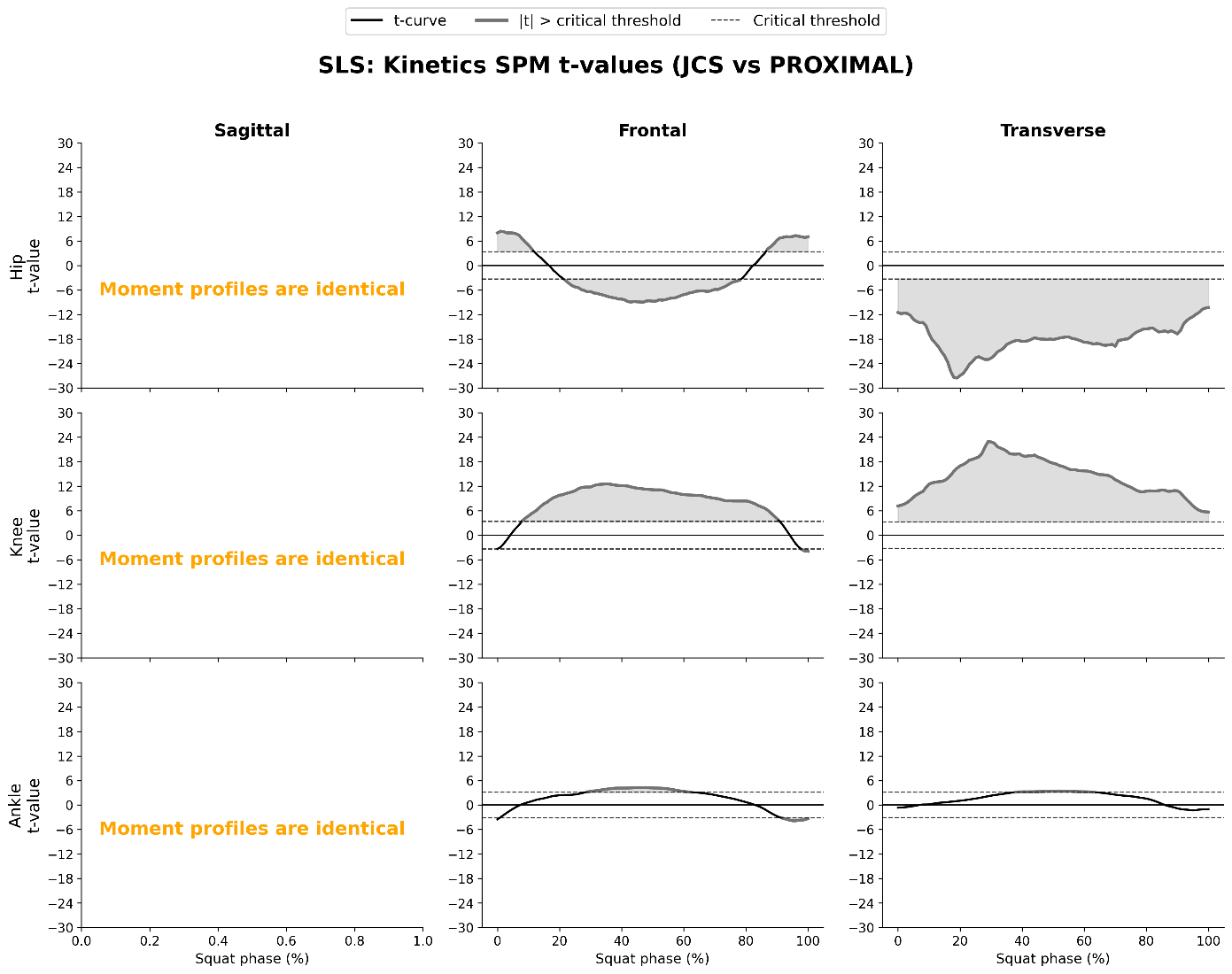


Supplementary Fig. S4. Comparison of CGM2.3 JCS and proximal coordinate systems on SLS.

# Appendix C

| **Variable** | **Upright position (mm)** | **Deepest position (mm)** |
| --- | --- | --- |
| **Thigh length CGM2.3** | 434.16 ± 24.45 | 434.16 ± 24.45 |
| **Thigh length PiG** | 412.72 ± 27.87 | 379.06 ± 24.92 |
| **Thigh length diff** | 21.44 ± 8.61 | 55.10 ± 18.98 |
|  |  |  |
| **Shank length CGM2.3** | 400.76 ± 22.13 | 400.76 ± 22.13 |
| **Shank length PiG** | 400.80 ± 21.84 | 370.44 ± 19.47 |
| **Shank length diff** | -0.04 ± 7.44 | 30.32 ± 12.85 |

Supplementary Table S3. Segment length differences of CGM2.3 and PiG in the SLS upright position and the deepest position of SLS. The peak values are presented as the average peak value. and the lowest and highest values are in parentheses. A positive value in the difference indicates a longer segment in CGM2.3. N = 72 squats.


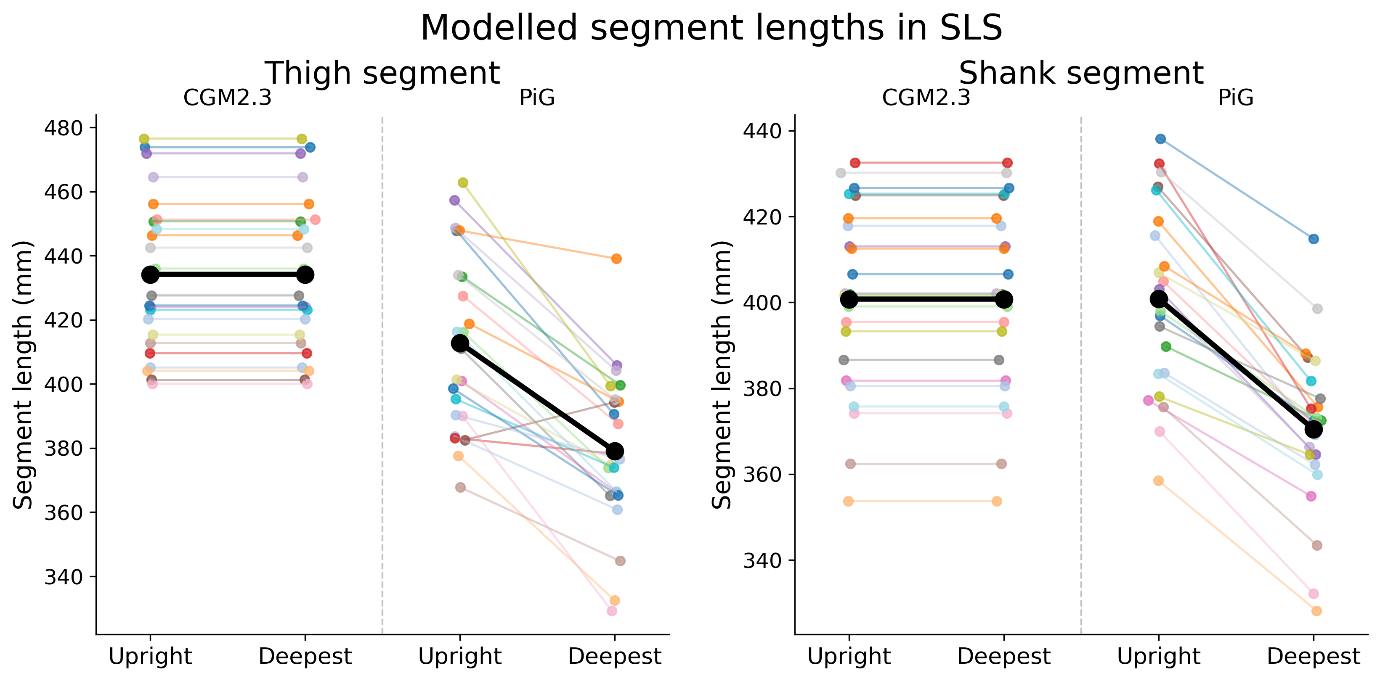


Supplementary Fig. S5. Segment length differences of CGM2.3 and PiG in SLS upright position and deepest position SLS. The values are presented as mean values from 3 squat averages at each position per subject. The black line presents the group mean. N = 72 squats.

# Appendix D

| **Joint** | **Metric** | **Upright position (mm)** | **Deepest position (mm)** |
| --- | --- | --- | --- |
| Hip | ΔX | 3.69 ± 3.99 | 13.16 ± 14.44 |
| Hip | ΔY | -3.31 ± 12.88 | 0.65 ± 13.43 |
| Hip | ΔZ | 17.19 ± 5.02 | 19.94 ± 8.77 |
| **Hip** | **d** | **22.33 ± 5.45** | **30.67 ± 9.52** |
|  |  |  |  |
| Knee | ΔX | -0.98 ± 7.33 | -40.02 ± 10.05 |
| Knee | ΔY | 0.96 ± 1.85 | 1.94 ± 3.99 |
| Knee | ΔZ | -3.53 ± 6.51 | -1.03 ± 10.80 |
| **Knee** | **d** | **9.92 ± 4.65** | **41.81 ± 9.72** |
|  |  |  |  |
| Ankle | ΔX | -6.01 ± 3.45 | -9.54 ± 6.79 |
| Ankle | ΔY | 0.67 ± 1.33 | 7.88 ± 4.83 |
| Ankle | ΔZ | -4.19 ± 3.33 | -15.36 ± 4.20 |
| **Ankle** | **d** | **8.67 ± 2.81** | **20.68 ± 7.08** |

Supplementary Table S4. Joint center location differences between CGM2.3 and PiG in the upright position and deepest position of the SLS. Values are expressed in global coordinates, where X = anteroposterior, Y = mediolateral, and Z = superior-inferior. Signed coordinate differences were calculated as CGM2.3 minus PiG and are reported as ΔX, ΔY, and ΔZ. The Euclidean distance between corresponding joint centers is reported as d. Positive signed values indicate higher coordinate values in CGM2.3 than in PiG. N = 72 squats.


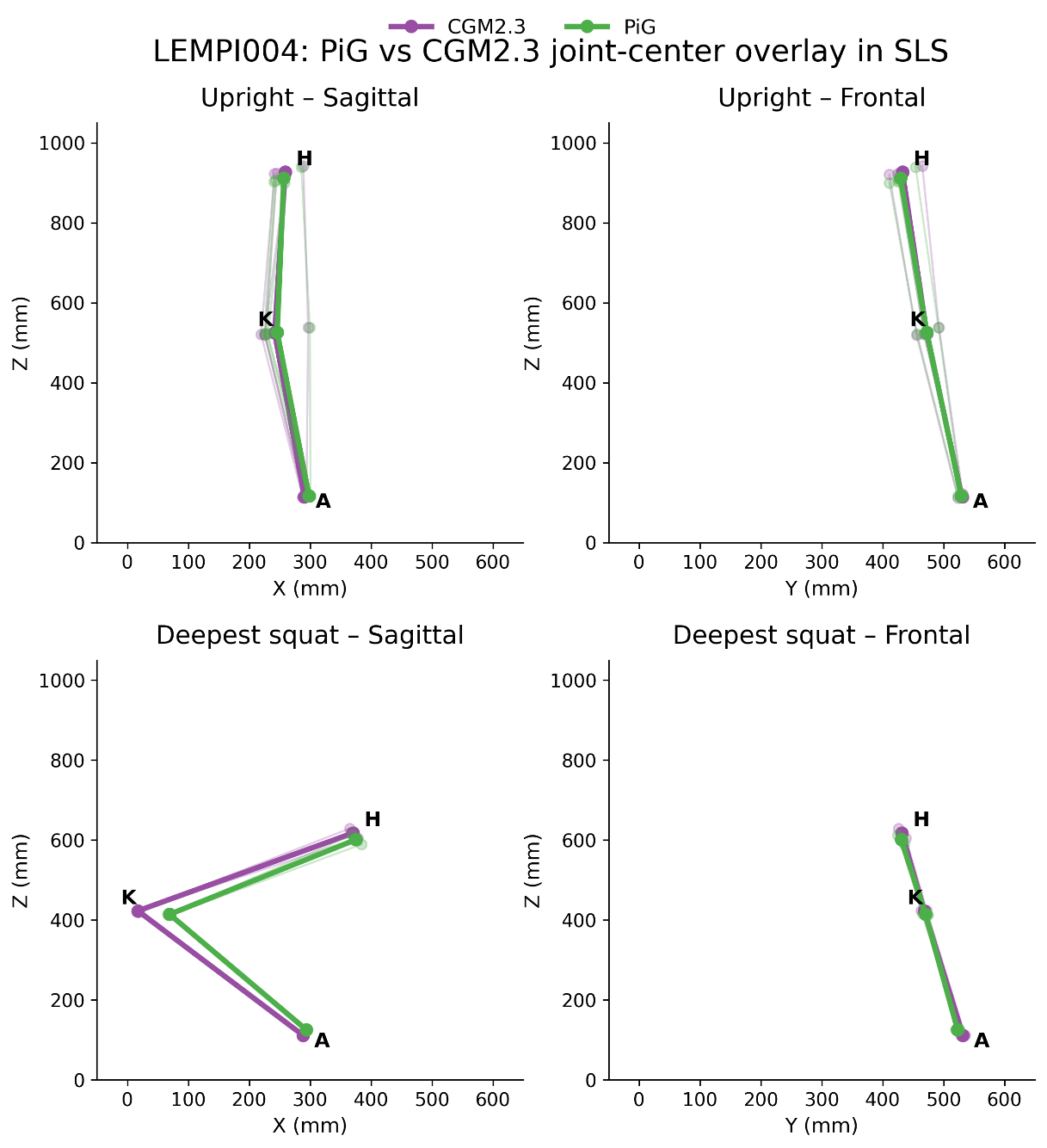


Supplementary Figure 6. Joint center positions of CGM2.3 and PiG in the upright and deepest positions of the SLS. Joint center locations and segment orientations are shown for a representative participant. selected as the individual closest to the group median based on the mean Euclidean distance (d) of the hip. knee. and ankle joint centers in the deepest squat position. Joint center positions were extracted from the same frame for both models. Background shading illustrates individual squats. while the solid representation indicates the mean position across three squats for that participant. N = 3 squats.
